# Radiograph Interpretation Discrepancies in a Community Hospital Emergency Department

**DOI:** 10.5811/westjem.2019.1.41375

**Published:** 2019-07-02

**Authors:** Michael J. Tranovich, Christopher M. Gooch, Joseph M. Dougherty

**Affiliations:** Ohio Valley Medical Center, Department of Emergency Medicine, Wheeling, West Virginia

## Abstract

**Introduction:**

In many hospitals, off-hours emergency department (ED) radiographs are not read by a radiologist until the following morning and are instead interpreted by the emergency physician (EP) at the time of service. Studies have found conflicting results regarding the radiographic interpretation discrepancies between EPs and trained radiologists. The aim of this study was to identify the number of radiologic interpretation discrepancies between EPs and radiologists in a community ED setting.

**Methods:**

Using a pre-existing logbook of radiologic discrepancies as well as our institution’s picture archiving and communication system, all off-hours interpretation discrepancies between January 2012 and January 2015 were reviewed and recorded in a de-identified fashion. We recorded the type of radiograph obtained for each patient. Discrepancy grades were recorded based on a pre-existing 1–4 scale defined in the institution’s protocol logbook as Grade 1 (no further action needed); Grade 2 (call to the patient or pharmacy); Grade 3 (return to ED for further treatment, e.g., fracture not splinted); Grade 4 (return to ED for serious risk, e.g., pneumothorax, bowel obstruction). We also recorded the total number of radiographs formally interpreted by EPs during the prescribed time-frame to determine overall agreement between EPs and radiologists.

**Results:**

There were 1044 discrepancies out of 16,111 EP reads, indicating 93.5% agreement. Patients averaged 48.4 ± 25.0 years of age and 53.3% were female; 25.1% were over-calls by EPs. The majority of discrepancies were minor with 75.8% Grade 1 and 22.3% Grade 2. Only 1.7% were Grade 3, which required return to the ED for further treatment. A small number of discrepancies, 0.2%, were Grade 4. Grade 4 discrepancies accounted for two of the 16,111 total reads, equivalent to 0.01%. A slight disagreement in finding between EP and radiologist accounted for 8.3% of discrepancies.

**Conclusion:**

Results suggest that plain radiographic studies can be interpreted by EPs with a very low incidence of clinically significant discrepancies when compared to the radiologist interpretation. Due to rare though significant discrepancies, radiologist interpretation should be performed when available. Further studies are needed to determine the generalizability of this study to EDs with differing volume, patient population, acuity, and physician training.

## INTRODUCTION

In many emergency departments (ED) across the United States and throughout the world, plain radiographic studies are initially interpreted by an emergency physician (EP) without the immediate interpretation of a trained radiologist. While EP interpretation aids in ED flow and prompt treatment, interpretation errors can potentially place a patient at unnecessary risk of adverse outcome and the treating physician at risk for litigation.^1^,^2^ Studies in the literature have suggested that immediate radiology interpretation has potential to reduce errors that would require call-back to the ED.^3^,^4^

Several studies have examined radiologic interpretation discrepancies between EPs and radiologists.^5^–^18^ These studies report a wide range of agreement between the two specialties in regard to plain radiograph interpretation in the ED. Two existing studies found agreement rates as high as 97–99% between EPs and radiologists.^15^,^16^ In contrast, other studies have found agreement rates as low as 52–66%.^5^,^7^ The large variability in the reported range of radiographic interpretation discrepancies suggests the need for further study in relation to this clinically relevant topic encountered on a day-to-day basis in many EDs without 24-hour radiologist coverage.

The aim of this study was to identify the number of radiologic interpretation discrepancies between EPs and radiologists in a community ED setting. We also sought to determine the frequency and nature of treatment- changing discrepancies to determine whether plain radiographs can be safely interpreted by EPs without immediate radiologist interpretation. A secondary aim was to examine the agreement in radiology reads based on age as well as the specific body area imaged. We hypothesized that radiographs interpreted by EPs would have a high level of agreement with final radiologist interpretation. Furthermore, we hypothesized that discrepancies presenting serious risk to the patient would be rare.

## METHODS

The setting for this study was a community hospital ED with an emergency medicine (EM) residency program in which “off-hours,” plain radiographs are not read by a radiologist until the following morning. These off-hours are generally 5–6 PM to 6:30 AM. The number of hours without radiologist coverage of plain radiographic reads varies per day based on the radiologist shift schedule, which generally ranges from 8–12 hours without coverage of radiograph interpretation. During times without radiologist coverage, initial interpretations, determined by the attending, board-certified EP, are logged into our picture archiving and communication system (PACS) and a board-certified general radiologist provides final interpretations in the morning.

Radiographic discrepancies are charted in the morning after radiology read in a discrepancy logbook by the day-shift attending EP. The degree of discrepancy, as explained below, is also determined and documented by the attending physician. Necessary callbacks are made by nursing based on the clinical judgment of the documenting, not interpreting, EP. No formal training is specifically given to the EP on discrepancy grading, although the grading follows straightforward guidelines. If in the clinical judgment of the documenting EP, a callback or return to ED was warranted this was directly reflected in the discrepancy grading.

Degree of discrepancy is graded on a 1–4 scale. Grade 1 is a minor discrepancy with no additional action needed, eg, an infiltrate read on radiologist interpretation of chest radiograph (CXR) but not seen overnight by the EP even though the patient was started on an antibiotic at the time of service. Minor “over-calls” by the EP that were not appreciated in the final radiology interpretation would also be considered Grade 1. For example, an EP interprets, “questionable fracture,” and instructs the patient to follow-up although the radiologist interprets, “no fracture.” Grade 2 is a minor discrepancy in which the patient was contacted and did not require return to the ED. For example, a radiologist interprets infiltrate on CXR, not appreciated on overnight read, which required an antibiotic to be called in to the pharmacy. An additional example would be informing a patient via phone call regarding a pulmonary nodule that was appreciated by the radiologist, which requires primary care follow-up. Grade 3 is a major discrepancy in which the patient was required to return to the ED for further treatment. An example would be calling a patient back to the ED to splint a fracture that was not appreciated on overnight EP read. Grade 4 discrepancies are major discrepancies that risk serious harm to the patient. Examples are a missed pneumothorax, free air under the diaphragm, small bowel obstruction, etc.

**Population Health Research Capsule**

What do we already know about this issue?

Existing studies have found conflicting agreement rates between emergency physicians (EP) and radiologists regarding plain radiograph interpretations.

What was the research question?

Can EPs accurately interpret plain radiographs without the immediate aid of a radiologist?

What was the major finding of the study?

EPs can accurately interpret commonly obtained radiographs with a very low rate of treatment-changing misses.

How does this improve population health?

While EPs interpret common films well, this study can improve emergency department care by stimulating EPs to be more cautious interpreting pediatric and less common radiographs.

Following institutional review board approval, we retrospectively reviewed all radiologic discrepancies recorded in our logbook and PACS between January 1, 2012 and January 1, 2015, regardless of patient age, gender, or presenting complaint. No discrepancies were excluded. Age and gender of the patient were documented. We also recorded the initial diagnosis, final diagnosis, body area of radiographic study, nature of discrepancy, grade of discrepancy, modified treatment, and disposition. The total number of EP radiographic reads during the time period studied were obtained from our PACS to determine overall EP and radiologist agreement. In addition, we queried our PACS and categorized EP reads by body area to determine if certain types of radiographs had a higher or lower level of agreement in interpretation. Radiographs were separated into groups as follows: chest, abdomen, lower extremity, upper extremity, cervical spine, thoracic-lumbar-sacral-coccygeal spine, pelvis, soft tissue neck, or other. Upper extremity included any radiograph performed at the level of the shoulder or distal to the shoulder. Lower extremity included any radiograph at the level of the hip or distal to the hip. The category of “other” included radiographs of the scapula, clavicle, sternum, nose, face, orbits, mandible, and ribs. We further categorized patients into the age groups of 0–6 years, 7–12 years, 12–17 years, and 18 years or greater to determine if discrepancy rates were higher in a particular age group.

Data were entered into, organized, and analyzed with PASW statistics (version 17.0, SPSS Inc., Chicago, IL). We determined frequency counts for all categorical variables, and measures of central tendency and dispersion were performed on continuous variables. Agreement percentages were calculated as 100 - [(number of discrepancies/number of reads) × 100].

## RESULTS

In our ED between January 2012 and January 2015, 16,111 radiographs were interpreted by an EP without the aid of immediate radiologist interpretation. Of these interpretations, there were 1044 discrepancies indicating an overall 93.5% agreement rate between EP and radiologist. The average age of patients with radiographic discrepancies was 48.4 ± 25.0 years, and 53.3% of patients were female. The age of patients with discrepancies ranged from 0.03 years to 98.3 years. Of patients with radiographic discrepancies, 28.8% were admitted to the hospital from the ED.

The majority of discrepancies, 75.8%, were very minor and required no further action after radiologist interpretation. Grade 2 discrepancies, which required a phone call to the patient, patient’s physician, pharmacy, and/or to the hospital floor accounted for 22.3% of discrepancies. Less than 2% of discrepancies required the patient to return to the ED and/or risked serious harm to the patient (Table 1). Based on the total number of EP radiology interpretations, discrepancies in radiograph interpretation led to 20 ED return visits, which is approximately 0.1% of the entire cohort studied. Based on the total 16,111 interpretations, Grade 1 discrepancies occurred in 4.9%, Grade 2 in 1.4%, Grade 3 in 0.1%, and Grade 4 in 0.01%.

Both of the Grade 4 discrepancies encountered in this study were small pneumothoraces. Patient 1 had a 3.5 centimeter (cm) pneumothorax as well as a rib fracture interpreted on rib radiographs by the radiologist that were not appreciated by the EP. This patient was previously discharged from the ED and required callback for further treatment. No additional significant morbidity was encountered as a result of the missed pneumothorax based on review of the patient’s hospital course. Patient 2 had a CXR performed in the ED that was initially interpreted as “effusion” by the EP. On radiology interpretation in the morning, a 2.5 cm pneumothorax was appreciated. This patient was admitted following his ED course; therefore, the medicine team was contacted and prompt surgical consultation was initiated. This patient experienced no obvious significant morbidity due to the missed pneumothorax.

Of the 1044 discrepancies studied, 66.6% were attributed to findings that were not originally appreciated by the EP at the time of service. Over-calls by the EP accounted for 25.1% of discrepancies, while 8.3% of discrepancies were based on a conflict in finding between the EP and radiologist (Table 1). For example, an EP interprets pneumonia on CXR while radiologist interprets vascular congestion or vice versa. CXRs accounted for 45.1% of the total number of radiographs interpreted by an EP, followed by 20.9% lower extremity, 17.7% upper extremity, and 6.2% abdominal. The remainder of body areas accounted for 10% of the total number of radiographs interpreted. Analysis of the various body areas revealed greater than 90% agreement in all types of radiographs except for those grouped into the category “other.” Only 31 radiographs were interpreted in this “other” category, with an agreement rate of only 35.5% (Table 2).

Of the 695 abnormalities not appreciated by the EP, the most common were the following, in descending order: 22.9% infiltrate; 16.5% fracture; 15.7% pulmonary nodule; 10.9% extremity finding other than fracture; 9.9% nonspecific lung density. In terms of possible or definite missed fractures, they were well distributed throughout the body areas with the highest percentages found in the forefoot including fifth metatarsal (13.0%), scaphoid (8.7%), rib (7.8%), triquetral (6.1%), ankle (6.1%), and thoracic or lumbar fractures (6.1%). Of the 262 findings that were over-called by the EP, the most common were as follows, in descending order: 35.5% fracture; 33.6% infiltrate; 9.2% pulmonary vascular congestion; 7.3% other lung finding; 5.3% extremity finding other than fracture. In terms of the over-called fractures, they were well distributed with the most frequent being forefoot (15.1%), ankle (10.8%), distal radius (9.7%), metacarpals/phalanges (6.5%), and rib (6.5%).

In subgroup analysis based on categorized age, radiographs of patients 0–6 years of age had an agreement rate of 70.8%. Radiographs of patients 7–12 years had an agreement rate of 92.1%, patients 13–17 years 89.4%, and those aged 18 or greater had an agreement rate of 94.1% (Table 3). Of the discrepancies in patients aged 0–6 years, 60% were EP misses and 35.7% were EP over-calls, with the remainder being a conflict in read. Of the EP misses in those 0–6 years, 64.3% were possible or definite infiltrates. Of the EP over-calls, 72.0% were possible or definite infiltrates.

## DISCUSSION

In this study we sought to examine discrepancies in plain radiographic reads between EPs and radiologists over a three-year period in our community hospital ED. While similar studies have been performed, data adding to the existing body of evidence are necessary as there are conflicting reports in the literature with agreement rates as low as 52%^7^ and as high as 97%–99%.^15^,^16^ We hypothesized that agreement would be high between the specialties. Our results support this hypothesis as we found a 93.5% agreement rate out of 16,111 total radiographic reads. We also hypothesized that discrepancies that place a patient at significant risk would be rare. Again, our results support this hypothesis, as only 0.01% of the total 16,111 reads were deemed to place a patient at serious risk. In total, only 0.1% of the total reads were determined to require return to the ED for further treatment.

Similar studies at academic institutions have found results comparable to ours. In a 1996 study performed at two academic EDs, Nitowski et al. performed an analysis of 14,046 radiographic studies interpreted by EP attending and radiologist and found a 0.95% disagreement rate with only 0.2% of the total being of clinical significance.^15^ While our overall disagreement rate was higher at 6.5% we found a similar rate of serious discrepancies: 2/16,111 in our study, compared to 3/14,046 in Nitowski et al.^15^ In a 1990 study performed by Gratton et al. at an emergency medicine residency program, the radiographic error rate between various EPs, including residents, with radiologist interpretation was reported as 3.4% overall with 2.8% of the total being of clinical significance.^10^ A 2011 study also found a very low rate of major discrepancies requiring emergent treatment, 85/151,693 (0.056%). In total, the authors found 4605 discrepant studies out of 151,693 radiographs.^16^

The findings of these studies, in combination with our data, suggest that plain radiographs can be interpreted by EPs with a very low occurrence of discrepancies that would place a patient at serious risk. A potential caveat is that two of the above-mentioned studies are over 20 years old and were performed in a time period with lesser technology in relation to electronic PACS. A more recent study performed in Iran in 2014 studied 105 trauma CXRs and found identical interpretation between EPs and radiologists in 89.5% of cases.^18^ The authors reported subcategories for differing traumatic injuries and found that EPs and radiologists had an agreement rate of 99% for hemothorax and 98.1% for pneumothorax.^18^

On the contrary, other studies have found conflicting results with much higher discrepancy rates than reported in our study. In a 2009 study by Al Aseri, 312 CXRs were studied and a 34% disagreement rate was reported between EPs and radiologists.^5^ In a 2005 study examining the agreement in pneumonia diagnosis on CXR between EPs and radiologists, the authors reported only a 52.3% agreement rate when combining reads of pneumonia or possible pneumonia.^7^ Of the 817 CXRs the EP read as pneumonia or possible pneumonia, the radiologist read normal in 21.2%, and 26.5% were interpreted as a process different from pneumonia. The authors explicitly mentioned that neither the EP nor the radiologist was held to blame as CXR is prone to significant inter- and intra-observer variability, even among radiologists. The authors also noted that EPs have the benefit of making a diagnosis on clinical grounds rather than just a static image and, therefore, the EP treatment may have been appropriate.^7^ In a 2018 study performed in Switzerland the authors examined discrepancies in interpretations for various imaging modalities, and in subgroup analysis of radiographs they found a discrepancy rate of 17.9% with a clinically significant disagreement rate of 5.67%.^13^

In conflict with the above studies, our data suggest that discrepancy rates in EDs may be much lower than the rather high discrepancy rates reported by these three studies. Regardless, data reported by these studies should alert an EP that discrepancies do in fact occur; and to minimize risk to a patient, an EP must not discount the value of radiologist interpretation.

While we reported discrepancies for all types of radiographs, the majority were CXRs and musculoskeletal extremity films. Facial, sternal, clavicular, orbital, nasal, and rib radiographs are obtained much less frequently in our ED; only 31 total as a group were interpreted first by an EP and the overall interpretation agreement was extremely low at 35.5%. This is in stark contrast to the greater than 90% agreement for films of frequently encountered body areas. Gratton et al. found a 9% disagreement rate when looking specifically at facial films, while they found lower rates of disagreement in more frequently encountered radiographs.^10^ Despite the low subgroup sample size we believe this suggests that EPs must exercise caution when interpreting films in which experience is lacking as this may lead to an increasing number of interpretive errors. In addition, while extremities films and CXRs are commonly encountered by the EP, our results may suggest the need for a more broad radiographic education for EPs as additional imaging modalities may not always be available to the EP.

In adult EDs, pediatric radiographs are generally encountered less frequently than those of older teenagers and adults. In our study only 1186/16,111 radiographs were in patients 17 years and younger. Furthermore, only 1.5% of the 16,111 radiographs were in patients six years and younger. Given the fact that radiographs of the very young are a small subset of day-to-day practice in an adult ED, experience can play a factor in radiographic interpretative error. Our agreement rate for those six years and younger was 70.8%, which is approximately 20% less than any other age group studied. In addition, the vast majority of misses and over-interpretations in this age group were infiltrates on CXRs. This common finding in our study could suggest the need for better education regarding interpretation of pediatric CXRs.

In a 2010 study, Johnson and Kline studied the intra- and inter-observer reliability of radiologists, senior pediatric EPs, and junior pediatric EPs in interpreting pediatric CXRs in patients aged 1–4 years.. Even in these pediatric-trained EPs, interpretative variability was considerably higher than among pediatric radiologists.^19^ Our subgroup analysis indicates that adult EPs must be vigilant when interpreting radiographs in the very young patient as interpretative discrepancies are more likely to occur than in adults presenting to a non-pediatric ED.

## LIMITATIONS

This was a single-site study in a community hospital ED with a limited number of practicing EPs and radiologists. This could negatively influence the overall generalizability of the study to sites with a greater or lesser number of physicians with differing levels of experience and/or training. Also, we were unable to determine whether an EP’s or radiologist’s number of years in practice had any correlation with interpretation accuracy. While all EPs interpreting radiographs in this study were board-certified, this is an area that warrants future research with well-designed prospective studies. In addition, we were unable to obtain an accurate gauge of the total number of radiographs with serious pathology such as abdominal free air, pneumothorax, bowel obstruction, etc. that were correctly interpreted by the EP over the studied time period. While our ED evaluates patients with pneumothorax, perforated viscus, and small bowel obstructions on a regular basis, we were unable to ascertain whether or not the percentage of treatment-changing discrepancies would be influenced if a higher incidence of serious pathology were encountered during off-hours.

The retrospective nature of this study did lead to weaknesses that should be addressed in future studies. Based on the documentation method used in our logbook as well as in our PACS, we were unable to determine the number of true-positives vs true-negatives. Agreements were simply documented as no deficiency whether they were a positive or negative study. As such, more robust measures of inter-rater reliability such as the kappa coefficient could not be calculated. Also as mentioned above, we were unable to determine the influence of the raters’ experience due to the method in which the deficiencies were documented. Prospective studies comparing the interpretation of more than one EP with more than one radiologist could account for these limitations. Despite this, our findings as well as our limitations suggest that despite the study of this topic in the past, more research is needed.

While we used the radiologist interpretation as the gold standard of diagnosis, it should be mentioned that there are potential issues with this design. There was more than one radiologist interpreting images in this study and radiologists can differ in their opinion or interpretation. It has been found that overall error rates between experienced radiologists is potentially around 3–9% in a mixture of negative and positive plain radiograph studies.^20^–^23^ Studies have abstracted from these data that if normal studies were excluded, error rates between radiologists could be as high as 30% in a grouping of abnormal studies.^20^ While this could have influenced our discrepancy percentage in a positive or negative direction, we feel it is unlikely to have skewed the results by more than a few percentage points based on the potential 3–9% radiologist disagreement rate reported above. In addition, the EP is more likely to have clinical clues, eg, pinpoint tenderness over the distal radius leading to an EP over-call of “possible fracture” while a radiologist is only provided a small history without aid of physical examination. This can introduce bias, mainly in regard to discrepancies in which an EP over-calls a specific radiographic study. While this may contribute to patient safety in a positive manner, it could potentially lead to overtreatment with antibiotics, immobilization, etc.

Finally, radiologists at our institution are not blinded to the overnight EP interpretation, and therefore this potentially introduces bias to the radiologist interpretation. Unfortunately in our PACS, the EP interpretation appears immediately when a radiologist opens the radiographic study. In general, radiologists at our institution do attempt to formulate their own interpretation of a study before fully reviewing the EP interpretation to determine if there are any conflicts. A project with blinding of the radiologist to the EP interpretation warrants further study in the future to determine if the radiologist interpretation is actually influenced by EP read.

## CONCLUSION

We found that plain radiographic studies can be interpreted by EPs with a low incidence of clinically significant discrepancies when compared to the final radiologist interpretation. While conflicting reports exist regarding disagreement in plain radiographic reads between EPs and radiologists, our study suggests that discrepancy rates are most consistent with studies reporting lower rather than higher discrepancy rates. Although serious discrepancies were rare in our study, radiologist interpretation should be performed immediately when available to limit the small number of treatment-changing discrepancies that could potentially place patients at risk of adverse outcome. Furthermore, while we found greater than 90% agreement in the most commonly obtained radiographs in the ED, infrequently obtained radiographs such as facial and rib films had a very poor agreement rate among EPs and radiologists. Increasing discrepancy rates were also found in patients aged six and younger when compared to adults and older pediatric patients. In the best interest of patient care, an EP should be hesitant to make treatment decisions based on their interpretation of infrequently obtained radiographs as well as in the very young if immediate radiologist interpretation is not available. Future prospective studies are needed to determine the generalizability of this study to EDs with differing volume, patient population, acuity, and physician training.

## Figures and Tables

**Table 1:** Discrepancies in radiograph read (n=1044).

Not appreciated by EP (%)	66.6
Over-call by EP (%)	25.1
Conflict in read (%)	8.3
Discrepancy grade (%)	
Grade 1	75.8
Grade 2	22.3
Grade 3	1.7
Grade 4	0.2

*EP,* emergency physician.

Grade 1 - No action needed; Grade 2 - Call to patient or pharmacy; Grade 3 - Major discrepancy, return to ED; Grade 4 - Major discrepancy, serious risk to patient.

**Table 2:** Radiographs and discrepancies by body area (n=16111).

Type of radiograph read by EP	% of total interpretations
Chest	45.1
Lower extremity	20.9
Upper extremity	17.7
Abdominal	6.2
TLS spine	5.2
Pelvis	2.5
Cervical spine	1.7
Soft tissue neck	0.4
Other	0.2
Discrepancy by radiograph type	# discrepancies/# reads (% Agreement)
Chest	616/7261 (91.5)
Lower extremity	165/3371 (95.1)
Upper extremity	135/2858 (95.3)
Abdominal	50/999 (95.0)
TLS spine	19/844 (97.8)
Pelvis	25/400 (93.7)
Cervical spine	8/278 (97.1)
Soft tissue neck	6/69 (91.3)
Other	20/31 (35.5)

*EP*, emergency physician; *TLS*, thoracic, lumbar, and/or sacrum coccyx; *#*, number.

Other includes scapula, clavicle, sternum, nasal, facial, orbital, mandible, ribs.

**Table 3:** Radiographic discrepancies by patient age (n=16111).

Age categorized	# discrepancies/# reads (% Agreement)
0 to 6 years	70/240 (70.8)
7 to 12 years	37/467 (92.1)
13 to 17 years	51/479 (89.4)
18 years and greater	886/14925 (94.1)

*#*, number.
